# Automated mapping of land cover in Google Earth Engine platform using multispectral Sentinel-2 and MODIS image products

**DOI:** 10.1371/journal.pone.0312585

**Published:** 2025-04-07

**Authors:** Xia Pan, Zhenyi Wang, Gary Feng, Shan Wang, Sathishkumar Samiappan

**Affiliations:** 1 College of Resources and Environmental Economics, Inner Mongolia Industrial Development Research Base, Inner Mongolia University of Finance and Economics, Hohhot, Inner Mongolia, China; 2 College of Statistics and Mathematics, Inner Mongolia University of Finance and Economics, Hohhot, Inner Mongolia, China; 3 Crop Science Research Laboratory, USDA Agricultural Research Service, Starkville, Mississippi State, United States of America; 4 Department of Discipline Construction, Inner Mongolia University of Finance and Economics, Hohhot, Inner Mongolia, China; 5 Geosystems Research Institute, Mississippi State University, Starkville, Mississippi State, United States of America; Universiti Kebangsaan Malaysia, Malaysia

## Abstract

Land cover mapping often utilizes supervised classification, which can have issues with insufficient sample size and sample confusion, this study assessed the accuracy of a fast and reliable method for automatic labeling and collection of training samples. Based on the self-programming in Google Earth Engine (GEE) cloud-based platform, a large and reliable training dataset of multispectral Sentinel-2 image was extracted automatically across the study area from the existing MODIS land cover product. To enhance confidence in high-quality training class labels, homogeneous 20 m Sentinel-2 pixels within each 500 m MODIS pixel were selected and a minority of heterogeneous 20 m pixels were removed based on calculations of spectral centroid and Euclidean distance. Further, the quality control and spatial filter were applied for all land cover classes to generate a reliable and representative training dataset that was subsequently applied to train the Classification and Regression Tree (CART), Random Forest (RF), and Support Vector Machine (SVM) classifiers. The results shows that the main land cover types in the study area as distinguished by three different classifiers were Evergreen Broadleaf Forests, Mixed Forests, Woody Savannas, and Croplands. In the training and validation samples, the numbers of correctly classified pixels under the CART without computationally intensive were more than those for the RF and SVM classifiers. Moreover, the user’s and producer’s accuracies, overall accuracy and kappa coefficient of the CART classifier were the best, indicating the CART classifier was more suitable to this automatic workflow for land cover mapping. The proposed method can automatically generate a large number of reliable and accurate training samples in a timely manner, which is promising for future land cover mapping in a large-scale region.

## 1. Introduction

Over the past few decades, satellite remote sensing has undergone dramatic changes in the revisit cycle, data quality, area coverage, and spatial resolution [1]. Extensive studies have strongly proved that remote sensing imagery is the best to monitor land cover information over a larger area [2]. Satellite remote sensing has been widely used as an effective and efficient means to monitor land cover patterns at a large geographic extent. Many researchers used LiDAR derived information, including DSM, DEM, point density and spatial statistics calculated from LiDAR data for distinguishing land cover types. More recently, Sentinel-2 satellites were launched in 2015 and 2017 with even finer spatial resolution and revisit times. The increase in data at fine resolutions and in time increases the potential benefit of algorithms to incorporate evidence from large numbers of satellite images into useful maps for monitoring landscape changes.

Classification is one of the most vital phases for extract land cover information, and the classification algorithms learned from the training samples should be extended to the whole imagery [3]. The machine learning classification algorithms are grouped into two categories: unsupervised and supervised [4–5]. The former aggregates the pixel characteristics of images in classes by analyzing the similarity of attributes, without the analyst’s contributions [6]. However, unsupervised classification requires rich-experienced operator input manually to improve the result accuracy [5,7]. By contrast, supervised classifications are generally considered superior to unsupervised ones in terms of operating procedure and result accuracy [8,9].

The supervised classification requires sufficient and good representative samples which are commonly selected and labeled by visual inspection or field survey [10,11]. The process of collecting representative samples is extremely time-consuming and labor-intensive. Although partly researchers tried to only label the most uncertain samples by optimizing machine learning methods in semi-supervised classification to improve the process of sample selection, the accuracy of the classification results is low and still needs further to be improved by selected and labeled samples manually [12–15]. To the best of our knowledge, the index-based calculation is one of the most commonly used methods for automatically extracting samples, however, the classification results are commonly rough, and the land cover types are far less than in nature [16]. Therefore, developing an automatic extraction method of samples and establishing the large-scale detailed sample dataset has great practical significance. In land cover information extraction, the traditional algorithms with the improved resolutions and kinds of remote sensing satellite have several problems, such as the inability to be applied to multispectral and hyperspectral satellite imagery, the weak generalization ability of the model and the difficulty of automating the construction of a training database. To solve these problems, the machine learning algorithm is necessary to be further improved. Farahmand et al. [17] evaluated the capability of various nonlinear regression models based on optical Sentinel-2 remote sensing images to estimate soil salinity. Their evaluation results confirmed that nonlinear regression models are superior to linear regression models in soil salinity estimation. Taghizadeh-Mehrjardi et al. used machine learning algorithms to predict the soil particle size fraction, and found that the ant colony optimization (ACO) had a higher accuracy [18]. Xu et al (2021) used Random Forests and ResNets classifiers to map eight metropolitan areas, comparing training areas drawn by different or consistent interpreters, and data splitting strategy using rules that allow or reduce spatial autocorrelation. They found large discrepancies among results built from crowdsourced training areas digitized by different experts; improving the consistency of labels can lead to substantial improvements in local climate zone classification accuracy [19]. Although there has been increasing attention toward land cover classification, most research has focused on investigating classifiers and methods, whereas limited attention has been placed on the application of training data. Training areas are polygons manually digitized by experts from very-high-resolution imagery to represent land cover classes, which are then used as reference geometries in sampling data from satellite imagery for model training and evaluation [19]. The process of identifying training areas is time-consuming and requires that experts have knowledge specific to the land cover scheme and cities of interest. High-quality training areas are the basis for generating training data for land cover mapping. To decentralize training area collection, the MCD12Q1 V6 product provides global land cover types at yearly intervals (2001-present) derived from different classification schemes in GEE.

The GEE cloud-based platform is an emergent technology with functionalities of an automatic parallel processing and fast computational platform the effective self-programming of remote sensing images, helping scientists to develop algorithms with less effort than before [20–23]. For instance, Gorelick et al. [20] showed that the GEE is a cloud-sharing platform at the planetary scale that provides Google’s massive computational capabilities for a variety of high-impact societal issues, including land cover classification, water management, drought monitoring, food security, and environment protection. Furthermore, according to Hansen et al. [24], it only took 100 hours to process 654,178 Landsat-7 images (about 707 terabytes) and produce a global forest distribution map in the GEE platform. This was reported as a valuable achievement because this process would have taken a million hours to complete without GEE [25–27].

To accomplish automatic classification of land cover types, the present study investigated the samples of multispectral Sentinel-2 images that were extracted and collected automatically from the Annual International Geosphere-Biosphere Programme (IGBP) classification scheme of the MODIS Version 6 Land cover Type 1 Product (MCD12Q1 V6 LC_Type 1). Through this scheme, a large-scale detailed sample dataset was established, and 70% of the samples selected from MCD12Q1 were used to train three state-of-the-art supervised classification algorithms, RF, CART, and SVM, which are the internal machine learning algorithms in the GEE. Additionally, spectral indices from multispectral Sentinel-2 Level-2A data that respond to different land cover types parameters: NDVI, NDWI, and NDSI, were used as classification features. All data used in this study were downloaded and processed through the GEE JavaScript API (Application Programming Interface). The efficient and open-access image analysis workflow provides a fast and reliable method to remotely map land cover types in the study area. The following are the unique contributions of this research:

a. A sample dataset with detailed classification categories of multispectral Sentinel-2 image is set up by the automatic labeling of samples from the IGBP classification scheme of MCD12Q1 V6 LC_Type 1.
b. Presented the differences of land cover types in automatic labeled Sentinel-2 database under the three most commonly used supervised classification algorithms, and a classifier that is most suitable for the above automatic workflow is proposed.
c. Examine the superiority of the GEE cloud platform to the method proposed in this paper. Multispectral Sentinel-2 and MODIS images can be easily processed and fast computed using this platform before without paying too much attention to the basic works.

## 2. Study area and datasets

### 2.1 Study area

**Fig 1:**
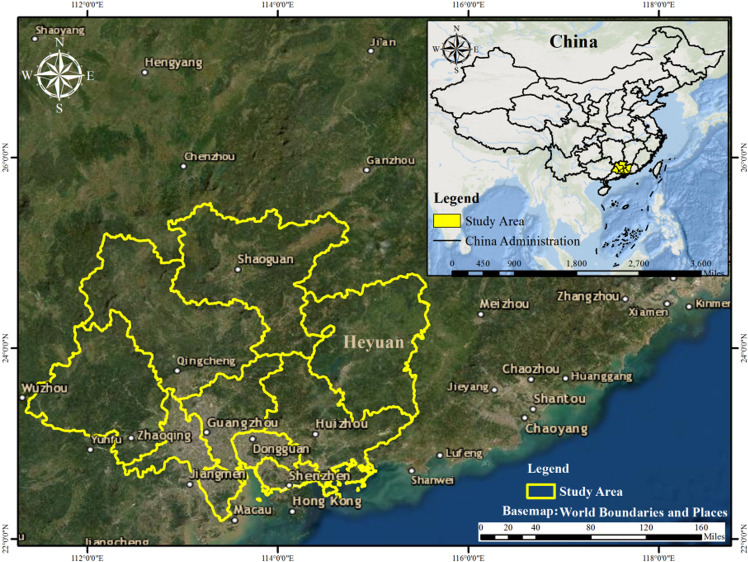
The study area with Guangdong province.

The study area with Guangdong province (E109°45′-117°20′, N20°09′-25°31′) which includes Shaoguan, Heyuan, Huizhou, Shenzhen, Dongguan, Guangzhou, Qingcheng, Zhaoqing, Foshan, Zhongshan cities is about 96205.45 km^2^ (Fig 1). The altitude ranges from 26 m to 1636 m above sea level. The average annual rainfall is from 1300 mm to 2500 mm, and the average air temperature is 22.3 °C. The main land cover types in Guangdong Province include Forest, Cropland, Urban and built-up areas and Water bodies. The chief reasons for choosing this study area are the Sentinel-2 image acquired has little cloud coverage and the vegetation types are complex and mixed. Consequently, the varied and visible land cover makes this area a good location for testing the ability of detailed and automated classification to sharpen land cover types that may be misidentified in a coarse classification.

### 2.2 MODIS land cover product

**Fig 2:**
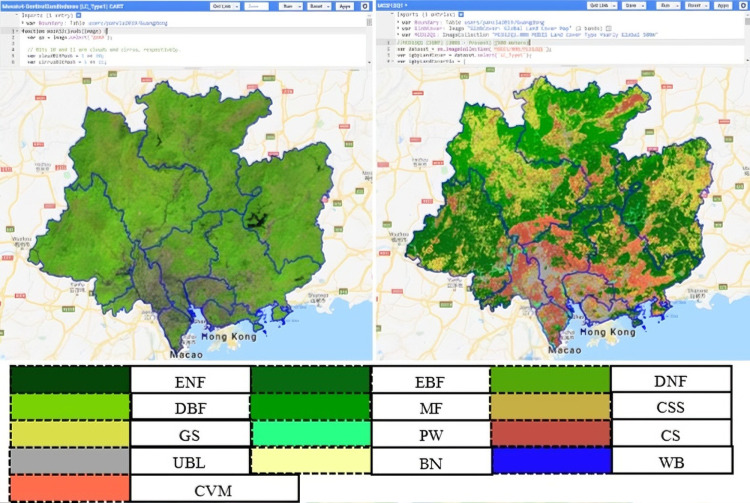
Sentinel-2 and MCD12Q1 for GEE cloud platform.

The MCD12Q1 V6 product provides global land cover types at yearly intervals (2001-present) derived from different classification schemes in GEE. The supervised classification of MODIS Terra and Aqua reflectance data was used. The supervised classification undergoes additional post-processing that incorporates prior knowledge and ancillary information to further refine specific land cover classes. LC_Type1 (17 classes) of MCD12Q1 from the International Geosphere-Biosphere Program (IGBP) was used to provide training class labels for Sentinel-based land cover classification. Detailed information refers to “https://lpdaac.usgs.gov/products/mcd12q1v006/”. The value, class names, abbreviations, and descriptions were summarized in Table 1. The clipped image and programming in GEE were shown in Fig 2 (Right).

**Table 1:** Value, class names, abbreviations, and descriptions of the IGBP classification scheme of MCD12Q1 V6 LC_Type 1.

Value	Class Name	Abbreviations	Description	CONUS area %
1	Evergreen Needleleaf Forests	ENF	Dominated by evergreen conifer trees (canopy> 2m). Tree cover > 60%	6.27
2	Evergreen Broadleaf Forests	EBF	Dominated by evergreen broadleaf and palmate trees (canopy> 2m). Tree cover > 60%	0.73
3	Deciduous Needleleaf Forests	DNF	Dominated by deciduous needleleaf (larch) trees (canopy> 2m). Tree cover > 60%	0.05
4	Deciduous Broadleaf Forests	DBF	Dominated by deciduous broadleaf trees (canopy> 2m). Tree cover > 60%	4.81
5	Mixed Forests	MF	Dominated by neither deciduous nor evergreen (40–60% of each) tree type (canopy> 2m). Tree cover > 60%	12.50
6	Closed Shrublands	CSS	Dominated by woody perennials (1-2m height) > 60% cover	0.38
7	Open Shrublands	OS	Dominated by woody perennials (1-2m height) 10–60% cover	12.53
8	Woody Savannas	WS	Tree cover 30–60% (canopy> 2m)	7.38
9	Savannas	SS	Tree cover 10–30% (canopy> 2m)	0.46
10	Grasslands	GS	Dominated by herbaceous annuals (<2m)	23.60
11	Permanent Wetlands	PW	Permanently inundated lands with 30–60% water cover and > 10% vegetated cover	0.64
12	Croplands	CS	At least 60% of area is cultivated cropland	14.31
13	Urban and Built-up Lands	UBL	At least 30% impervious surface area including building materials, asphalt and vehicles	1.23
14	Cropland/Natural Vegetation Mosaics	CVM	Mosaics of small-scale cultivation 40–60% with natural tree, shrub, or herbaceous vegetation	10.44
15	Permanent Snow and Ice	PSI	At least 60% of area is covered by snow and ice for at least 10 months of the year	0.02
16	Barren	BN	At least 60% of area is non-vegetated barren (sand, rock, soil) areas with less than 10% vegetation	1.24
17	Water Bodies	WB	At least 60% of area is covered by permanent water bodies	3.41

### 2.3 Sentinel-2 multispectral product

The Sentinel-2 Level-2A product (from 1 January 2019–31 December 2019), which is a wide-swath, high-resolution imaging mission supporting Copernicus Land Monitoring studies, was obtained in the GEE cloud-based platform. Sentinel 2 includes Level-2A and Level-2B satellites. The Sentinel-2 Level-2A product carries a Multispectral Instrument (MSI) with a flight altitude of 786 km, a ground swath width of 290 km, incorporating 13 spectral bands: Visible and Near Infrared (NIR) at 10 m spatial resolution; red edge and Short-wave Infrared (SWIR) at 20 m spatial resolution, and three atmospheric bands at 60 m spatial resolution (Table 2). The quality band at 60 m (Q60) spatial resolution was used to mask out clouds. The mosaic image and programming in GEE are shown in Fig 2 (Left). Please see the details in “https://sentinel.esa.int/documents/247904/685211/Sentinel-2_User_Handbook”.

**Table 2:** Details of the Sentinel-2 Multispectral Instrument.

Name	Scale	Pixel Size (m)	Wavelength (nm)	Description
B1	0.0001	60	443.9 - 442.3	Aerosols
B2	0.0001	10	496.6 - 492.1	Blue
B3	0.0001	10	560 - 559	Green
B4	0.0001	10	664.5 - 665	Red
B5	0.0001	20	703.9 - 703.8	Red Edge 1
B6	0.0001	20	740.2 - 739.1	Red Edge 2
B7	0.0001	20	782.5 - 779.7	Red Edge 3
B8	0.0001	10	835.1 - 833	Near Infrared (NIR)
B8A	0.0001	20	864.8 - 864	Red Edge 4
B9	0.0001	60	945 - 943.2	Water Vapor
B10	0.0001	60	1373.5 - 1376.9	Cirrus
B11	0.0001	20	1613.7 - 1610.4	Short-wave Infrared (SWIR 1)
B12	0.0001	20	2202.4 - 2185.7	Short-wave Infrared (SWIR 2)

The processing with scene classification and the atmospheric correction was applied to the Sentinel-2 Level-2A product. Atmospheric correction applied to Top-of-Atmosphere (TOA) Level-1C orthoimage products removed the effects of the atmosphere on the TOA reflectance values of original remote sensing images, and this process was accomplished by using a set of look-up tables generated by libRadtran which is a unitless measurement that provides the ratio between the radiation reflected and the incident solar radiation on a given surface. Further, the scene classification algorithm generates a classification map, which consists of four different classes for clouds (including cirrus), together with six different classifications for shadows, cloud shadows, vegetation, soils/deserts, water, and snow. The main output of the Sentinel-2 Level-2A product is an orthoimage Bottom-of-Atmosphere (BOA) corrected reflectance product. Further, the 20% cloud cover in Sentinel-2 Level-2A product was filtered to get less cloudy granules. Finally, the Sentinel-2 Level-2A images with minimal cloud/haze from 1, 2, 3, 4, 5, 6, 7, 8, 11 bands, and 12 of 10, 20, and 60 m spatial resolutions were used. Sentinel-2 Level-2A output images used in the present study were resampled to 20 m in order to generate an equal spatial resolution for all spectral bands.

## 3. Method

All the processes were performed in the GEE cloud platform. At first, a large and reliable training dataset of multispectral Sentinel-2 image was extracted systematically across the study area from 17 land cover classes of the IGBP classification scheme of MCD12Q1 V6 LC_Type 1 product (3.1 Section). Then, the quality control and spatial filter were applied for all the land cover classes in order to generate a reliable and representative training dataset (3.2 Section). Further, spectral reflectance indices were added to the feature inputs used for land cover classification and mapping (3.3 Section), the reliable training datasets were applied to train the SVM, CART, and RF classifiers, and a confidence map was produced (3.4 Section). Finally, the classification accuracy based on the confusion matrix was evaluated by using new independent layered validation samples (3.5 Section).

### 3.1 Automatic collection of training samples from MCD12Q1 land cover product

According to the earlier approach proposed by Hankui and David [28], the MCD12Q1 V6 and Sentinel-2 Level-2A images were resampled to the same spatial resolution with 20 m and transformed to the same map projection using Geographic Lat/Lon (EPSG: 4326). Then, multispectral Sentinel-2 images were classified to generate a new land cover map, based on the MODIS IGBP land cover classification scheme. In this process, to reduce the spectral variability of the 20 m pixels within the 500 m pixels caused by heterogeneous pixels, criteria were designed according to Xie et al. [29,30], whereby, all the 500 m MODIS pixels were homogeneous and all the 20 m Sentinel-2 pixels within the 500 m MODIS pixel were homogeneous. These criteria helped select only high-quality training class labels for which there was high confidence. The formulas used are described below:


$$\text{Spectral centroid:}\rho_{\text{c}}=median\left(\left\{{Ρ}_{i} |i=1,2,\ldots ,n\right\}\right)$$ (1)

$$\text{Euclidean distance: }\Delta_{\text{i}}=\sqrt{\sum_{j=1}^{6}{\left(\rho_{i}^{j}-\rho_{c}^{j}\right)}^{2}}$$ (2)

Where ρ_i_ was a vector that consisted of the reflectance values of the Sentinel-2 Level-2A image; ρ_c_ was the spectral centroid and was the median value of a sample set in the spectral dimension; ∆ _i_ was the Euclidean distance from sample i to the spectral centroid of the samples.

**Fig 3:**
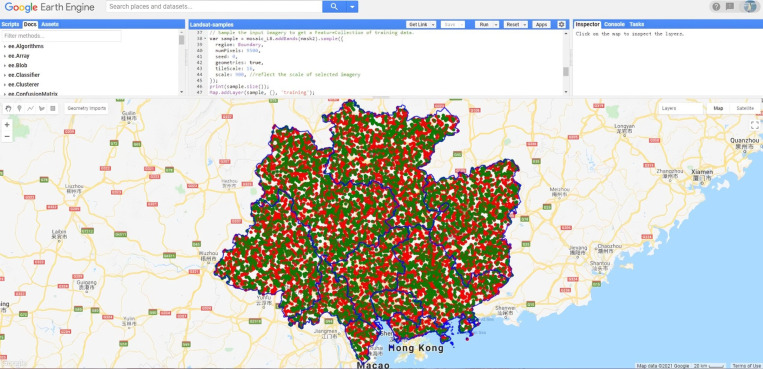
Automatic collection of training samples from MCD12Q1 (The red and green dots are training and verification samples respectively).

**Fig 4:**
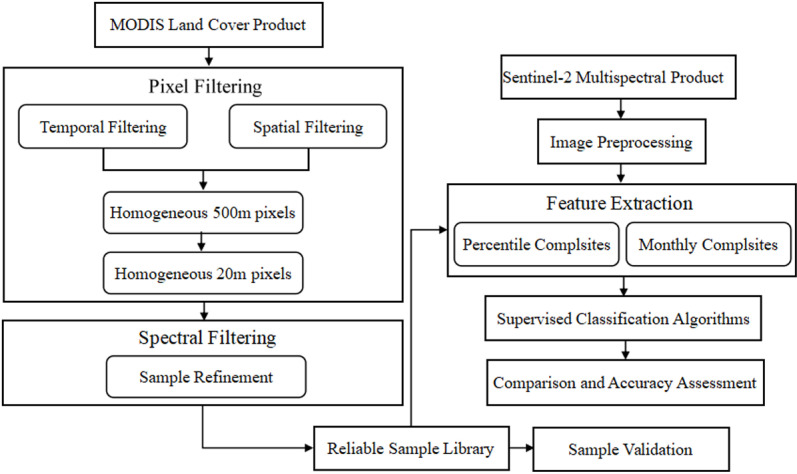
The main steps of the proposed method used in this study.

Then, we sorted the calculated Euclidean distance from small to large and retained the top 50% of the samples. Nearly 15% of heterogeneous 20 m pixels, which were inconsistent with the 500 m MCD12Q1 pixels, were removed by the above refinement process. Finally, a large and refined training dataset of multispectral Sentinel-2 image was extracted systematically and automatically across the study area from 17 land cover classes of the IGBP classification scheme of MCD12Q1 V6 LC_Type 1. The spatial distribution of samples and programming in GEE was shown in Fig 3. Flowchart showing the main steps of the proposed method used in this study was shown in Fig 4.

MCD12Q1 products were used to select accurate training samples automatically; the selected samples were then used to extract Landsat spectral-temporal features which were used to train the classifier. This process aimed to automatically generate land-cover mapping at 20 m using Sentinel data. The preliminary filtering criteria for MCD12Q1 products that were used are described below. These criteria were designed to help select only high-quality training class labels in which confidence was high:

(i) the MCD12Q1 pixels that had the same values in the 8 surrounding pixels;
(ii) the 500 m MODIS pixels were homogeneous;
(iii) the 20 m Sentinel pixels within the 500 m MODIS pixel were homogenous.

Rule (i) helped to reduce 500 m pixel edge effects in situations where there were possible changes in the underlying land cover. Rule (ii) was introduced because the homogeneous pixels had a higher classification accuracy. Rule (iii) helped to reduce the spectral variation within the 20 m pixels caused by heterogeneous pixels.

### 3.2 Quality control and spatial filter of land cover pixels

The quality of MCD12Q1 500 m land cover pixels was first considered to ensure the generation of a reliable and representative training dataset. Therefore, the suitable MCD12Q1 pixels that always had classification confidence (Land Cover_Type_1_Assessment) >  50% and quality assessment (Land Cover_Type_QC) set as “good quality” were selected [28]. According to the earlier approach proposed by Paula et al. [31], a spatial filter was also applied for all the land cover classes to reduce the edge effects and geolocation error caused by spatial differences between the 500 m MCD12Q1 and 20 m Sentinel-2 data, in which only the same land cover class in the surrounding eight MCD12Q1 500 m pixels was obtained. A minority of heterogeneous 20 m pixels which were inconsistent with the land cover types in 500 m MCD12Q1 pixels, were filtered using the above refinement process (Fig 4).

### 3.3 Spectral indices calculations in Sentinel-2 image data

Three spectral reflectance indices were added to the feature inputs 0used for further land cover classification.

Normalized Difference Vegetation Index (NDVI): NDVI is a commonly implemented index to assess vegetative growth (or biomass), drought, and agricultural production, based on surface information from multispectral measurements. NDVI is defined as (3):


$$\text{NDVI}=\frac{\text{NIR-Red}}{\text{NIR}+\text{Red}}$$ (3)

Normalized Difference Water Index (NDWI): NDWI uses the low reflectivity of water in the infrared band and high reflectivity in the green band to enhance the detection of water [32]. NDWI can effectively extract the water content of the vegetation canopy, and can also respond to the vegetation canopy under water stress in a timely manner, which is of great significance for drought monitoring, and is defined as (4):


$$\text{NDWI}=\frac{\text{Green-NIR}}{\text{Green}+\text{NIR}}$$ (4)

Normalized Difference Snow Index (NDSI): NDSI uses the high reflectivity in the visible and near-infrared bands and low reflectivity in the short-wave infrared band, which is the theoretical basis of remote sensing snow mapping, and is defined as (5):


$$\text{NDSI}=\frac{\text{SWIR-Red}}{\text{SWIR}+\text{Red}}$$ (5)

Where: NIR = near infrared band (Band 8); RED =  red band (Band 4); Green =  green band (Band 3); SWIR =  short-wave infrared (Band 11).

### 3.4 Supervised classification algorithms

In this study, 70% of the samples selected from MCD12Q1 were used to train SVM, RF, and CART classifiers which are the internal machine learning algorithms in the GEE cloud platform, and the remaining 30% of the samples were used for testing. Please refer to the official developer’s guide for the detailed application of the three above supervised classification algorithms used in this work (https://developers.google.com/earth-engine/guides/classification).

Typically, SVM classifiers analyze linearly separable cases. For linearly inseparable cases, the linearly inseparable samples in the low-dimensional input space are converted into high-dimensional feature spaces by using a nonlinear mapping algorithm to make them linearly separable, producing samples with nonlinear characteristics that can be analyzed linearly [3,33]. Moreover, the SVM classifier is based on the structural risk minimization theory in order to construct an optimal hyperplane in the feature space, so that the classifier is globally optimized, and the expectations in the entire sample space meet an upper bound with a certain probability [10,31]. The kernel trick is used to avoid the definition of the mapping function [34]. In the present study, the linear kernel was performed and the regulation parameter (C) was set to 102.

The RF algorithm generates multiple decision trees by creating random features, which combines Breiman’s idea of “Bagging” and random selection of features [35]. Three parameters need to be identified: the number of trees, the minimum number of terminal seeds, and the number of features [36]. Previous studies have indicated the accuracy becomes more stable if the number of trees is more than 120 [37,38] so the number of trees was set to 300 in this study. The other two parameters were adopted the default values (the minimum number of terminal seeds was 1, the number of features was the square root of the number of all features).

The CART classifier uses the binary recursive segmentation method with the Gini coefficient as the optimal test variance and segmentation threshold standard, and finally generates a classification decision tree based on the binary tree. The core idea of CART is to apply the attribute characteristics of the sample as test variables in the process of training data to generate a multi-level and multi-node binary tree. The classification process stops when there is no further split [31]. The complexity of the model was determined by the maximum depth of the tree. A large model depth may have greater accuracy, but it also increases the risk of overfitting. Qian et al. [39] found the overall accuracy becomes relatively stable when the maximum depth is set 5–8. Thus, the depth parameter in this study was set to 8.

### 3.5 Accuracy assessment

Accuracy was assessed using new independent layered validation samples, which were intuitively interpreted from the high-resolution images of the GEE cloud platform. Moreover, the “Bing Map”web service and System for Terrestrial Ecosystem Parameterization sites were also implemented, as well as the field photos from the Global Field Photo Library (http://www.eomf.ou.edu/photos/FieldPhoto/) [29]. In summary, a total of 5321 training samples and 3701 validation samples were extracted from the above datasets.

The traditional metrics, including user’s accuracy, producer’s accuracy, and overall accuracy, were used to quantitatively assess the classification accuracy. Although overall accuracy is correlated strongly with overall classification accuracy, it only computes the number of pixels correctly classified in the diagonal direction. Therefore, in addition to the pixel statistics on the diagonal, accuracy assessment also involved the Kappa coefficient, which calculates the missing and misclassified pixels outside the diagonal. Kappa is always between 0 and 1. Values with 0.80 to 1.00 imply very good agreement, 0.60 to 0.80 imply good agreement, 0.40 to 0.60 imply moderate agreement, 0.20 to 0.40 imply fair agreement, and less than 0.20 implies poor agreement. Given this, the user’s accuracy, producer’s accuracy, overall accuracy, and Kappa coefficient based on the confusion metrics were computed to comprehensively and quantitatively assess the proportion of correctly classified, missing, and misclassified pixels.

All the accuracy indices are presented with a 95% confidence interval. Finally, a land cover map was generated for study areas using the proposed method in this study and the classification accuracy of each map was computed using the corresponding validation datasets.

## 4. Results

### 4.1 The area distribution of different classifiers and land cover types

**Fig 5:**
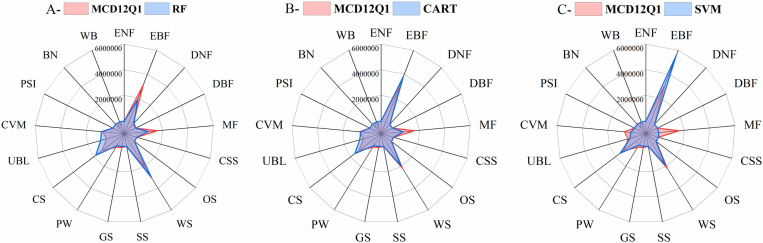
The area distribution of 17 different land cover types under MCD12Q1 and classifiers.

Fig 5 is the area distribution of different land cover types under MCD12Q1 and classifiers. The areas of the EBF, MF, and PW under the RF classifier were less than the MCD12Q1, however, the areas of the WS, CS, UBL, and CVM were greater than the MCD12Q1 (Fig 5A). The area distribution of land cover types under the CART classifier was similar to the RF, such as the areas of the MF and PW were still less than the MCD12Q1, the areas of the CS, UBL, and CVM were also more than the MCD12Q1 (Fig 5B). The area difference between the SVM classifier and MCD12Q1 was relatively large. The area of the EBF under the SVM classifier was greater than the MCD12Q1. Moreover, the areas of the MF and CVM were far less than the MCD12Q1 (Fig 5C). Therefore, among the three classifiers, the areas of different land cover types classified under CART were closest to the MCD12Q1.

**Fig 6:**
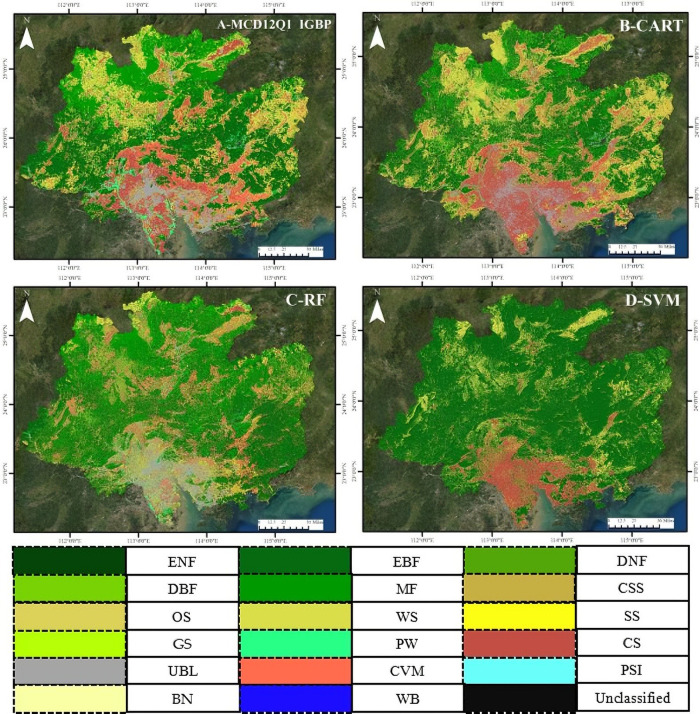
The spatial distribution of 17 different land cover types under MCD12Q1 and classifiers.

Fig 6 is the spatial distribution of different land cover types under MCD12Q1 and classifiers. The land cover types in the northern part of the study area under MCD12Q1 were mainly MF, WS, and a small part of CS and CVM. Moreover, large areas of EBF were mainly distributed in the eastern, western, and central parts of the study area. The southern part of the study area near the sea was dominated by UBL, surrounded by a large area of CS and CVM. Furthermore, there were scattered PW in the southwest (Fig 6A). The overall spatial distribution of the land cover types under the CART classifier was similar to MCD12Q1; however, CART has less area of EBF a more scattered spatial distribution of UBL. The land cover types in the northern part of the study area under the CART classifier were still mainly dominated by the MF, WS, and scattered CS and CVM. Compared with MCD12Q1 and to a lesser degree the RF classifier, there were no obvious areas of the PW under the CART classifier (Fig 6B and 6C). There were significantly more UBL near the southern part under the RF classifier than MCD12Q1. Moreover, the spatial distribution of WS under the RF classifier was quite different from MCD12Q1, and there was no obvious large-scale WS in the northern part. Although the area of EBF under the RF classifier was significantly more than that of MCD12Q1, it was still mainly distributed in the eastern, western, and central parts (Fig 6C). Compared with MCD12Q1, there was no obvious UBL under the SVM classifier in the southern part, and the spatial distribution of WS was also significantly different from MCD12Q1. Furthermore, there was no obvious PW under the SVM classifier (Fig 6D).

### 4.2 The pixel percentage of classifiers under each land cover type

**Fig 7:**
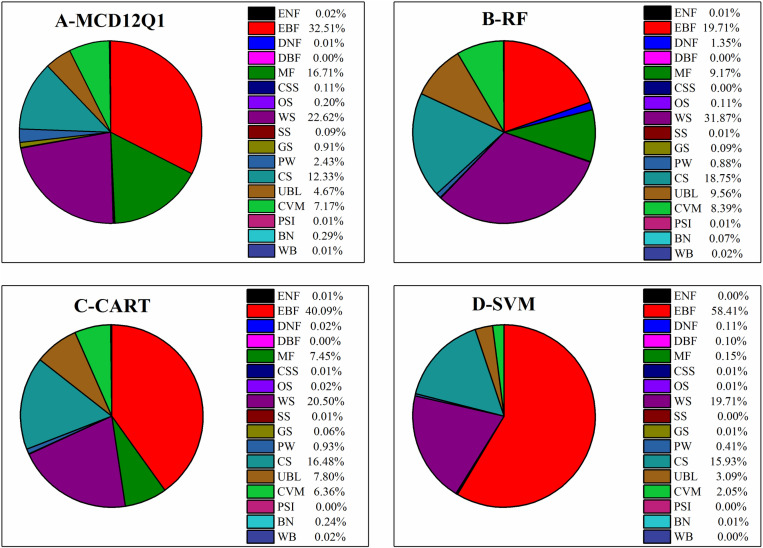
Pixel percentage of each classifier to the total under 17 different land cover types.

Fig 7 presents the pixel percentage of each classifier to the total pixels for the 17 land cover types. Under the MCD12Q1 classification system, the pixel percentages of the EBF reached 32.51% with the highest value for this system. Next, the pixel percentages of the WS, MF, CS were 22.62%, 16.71%, and 12.33%, respectively. The pixel percentages of the CVM and UBL were less and were only 7.17% and 4.67%. The pixel percentages of the other land cover types were even less and the total percentage was only 3.17% (Fig 7A). Under the RF classifier, the pixel percentage of the WS was obviously higher than the other land cover types and was 31.87%; however, the pixel percentage of the EBF was less than the MCD12Q1 system. Additionally, the pixel percentages of the EBF and CS were similar and reached 19.71%, 18.75%, respectively. The pixel percentages of the UBL, MF, and CVM were less and were 9.56%, 9.17%, 8.39%, respectively. Moreover, the pixel percentages of the CS, UBL, and CVM were visibly greater than the MCD12Q1 (Fig 7B).

Under the CART classifier, the pixel percentages of the EBF and MF were far different from the MCD12Q1, among which the pixel percentage of the EBF was more and the pixel percentage of the MF was less compared to the MCD12Q1. The pixel percentages of the WS and CVM were 20.50%and 6.36%, respectively, close to values under MCD12Q1. In addition, the pixel percentages of the CS and UBL were more than the MCD12Q1 and reached 16.48% and 7.80%, respectively (Fig 7C). The pixel percentages of different land cover types under the SVM classifier were far different from the MCD12Q1. The pixel percentage of the EBF was 58.41%, considerably more than MCD12Q1. The pixel percentages of the WS and CS were 19.71% and 15.93%. In addition, the pixel percentages of other land cover types were less and different from the MCD12Q1 (Fig 7D). In short, the main land cover types in the study area were the EBF, MF, WS, and CS under the four different classifiers. The pixel percentages for different land cover types were most similar under the CART and MCD12Q1 classifiers.

### 4.3 The accuracy assessment based on the confusion matrix

**Fig 8:**
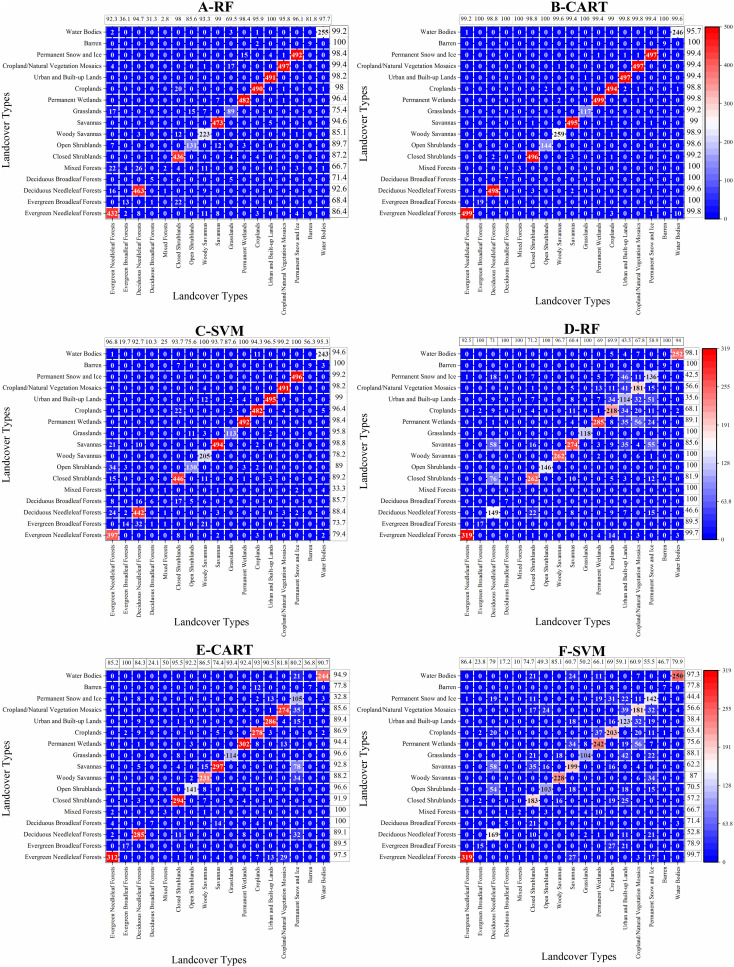
A-C and D-F correspond to the confusion matrix of the training samples and the validation samples respectively.

Fig 8 is the confusion matrix of training and validation samples. A-C is the confusion matrix of training samples, and D-F is the confusion matrix of validation samples. The total number of pixels for each land cover type in the study area was set at 500 for training samples and 320 for validation samples. Due to the small area of minority land cover types, the total number of pixels for minority land cover types failed to reach the set value (such as Evergreen Broadleaf Forests, Deciduous Broadleaf Forests, Mixed Forests, and Barren).

The number of correctly classified pixels of the Permanent Wetlands, Croplands, Urban and Built-up Lands, Cropland/Natural Vegetation Mosaics, and Permanent Snow and Ice under the RF classifier was all greater than 480. Next, the number of correctly classified pixels of Savannas, Closed Shrublands, Evergreen Needleleaf Forests, and Deciduous Needleleaf Forests was second only to the above land cover types and all types had greater than 400 pixels. The total pixel numbers of other land cover types were far less than 500, such as Evergreen Broadleaf Forests, Deciduous Broadleaf Forests, Woody Savannas, Grasslands, Open Shrublands, Mixed Forests, and Barren (Fig 8A). The number of misclassified pixels under the RF classifier was obvious, for example, 15 pixels in the Permanent Wetlands were misclassified into the Permanent Snow and Ice, 17 pixels in the Grasslands were misclassified into the Cropland/Natural Vegetation Mosaics, and there were 20 and 22 in the Closed Shrublands that were misclassified into the Croplands and Evergreen Broadleaf Forests, respectively (Fig 8A). Compared to the RF classifier, there were more land cover types for which the number of correctly classified pixels reached nearly 500 pixels under the CART. The number of correctly classified pixels of the Evergreen Needleleaf Forests, Deciduous Needleleaf Forests, Closed Shrublands, and Savannas under the CART classifier was more than the RF. Although the total pixel numbers of Deciduous Broadleaf Forests, Mixed Forests, and Barren under the CART classifier were only 7, 3, and 9, respectively, there were no misclassified pixels. Additionally, the total pixel numbers of the Open Shrublands, Woody Savannas, Grasslands, and Water Bodies were far less than the set value equal to 500 pixels, and the number of misclassified pixels under the CART classifier were less than the RF (Fig 8B). The number of correctly classified pixels under the SVM classifier was less than the CART and similar to the RF classifier. There was only the Barren without misclassified pixel in the SVM classifier. (Fig 8C). Further, in the training samples, the user’s and producer’s accuracies of the Savannas, Permanent Wetlands, Croplands, Urban and Built-up Lands, Cropland/Natural Vegetation Mosaics, Permanent Snow and Ice, and Water Bodies under the RF and SVM classifiers were similar and nearly the 100%. However, the user’s and producer’s accuracies of each land cover type under the CART classifier were almost the same and very close to 100%, indicating the accuracies of CART in the training sample were better than those in the RF and SVM classifiers.

The number of correctly classified pixels in the Evergreen Needleleaf Forests under the RF classifier was higher than the other land cover types in which the numbers of correctly classified pixels were less than 300, and were nearly the set value equal to 320 pixels. The total pixel number of the Deciduous Broadleaf Forests, Mixed Forests, and Barren were equal to the number of correctly classified pixels, indicating no misclassified pixels. There were obvious misclassified pixels of some land cover types under the RF classifier, and even the number of misclassified pixels was more than the correctly classified pixels. For example, the number of correctly classified pixels was 136 for the Permanent Snow and Ice; however, the number of misclassified pixels was 184, among which 55 and 51 pixels were misclassified into Savannas and Urban and Built-up Lands, respectively; the number of misclassified pixels of the Deciduous Needleleaf Forests were 171 pixels and more than the correctly classified pixels (149 pixels), among which 76 and 58 pixels were misclassified into the Closed Shrublands and Savannas, respectively (Fig 8D). The number of correctly classified pixels of the Evergreen Needleleaf Forests under the CART classifier was still the highest. Although there were inevitably misclassified pixels under the CART classifier, the number of correctly classified pixels among different land cover types was substantially above the number of misclassified pixels (except for the Permanent Snow and Ice). The CART classifier misclassified 215 pixels of the Permanent Snow and Ice into the Cropland/Natural Vegetation Mosaics, Urban and Built-up Lands, Savannas, Woody Savannas, Deciduous Needleleaf Forests, and Water Bodies (Fig 8E).

The number of misclassified pixels under the SVM classifier was more than the RF and CART classifiers, such as the Croplands, Permanent Wetlands, Savannas, and Closed Shrublands, etc. The number of correctly classified pixels of the Evergreen Needleleaf Forests was equivalent under the SVM and RF classifiers and reached the highest value (Fig 8F). Further, in the validation samples, the user’s and producer’s accuracies of the Deciduous Needleleaf Forests, Croplands, Urban and Built-up Lands, Cropland/Natural Vegetation Mosaics, and Permanent Snow and Ice under the RF classifier were low, ranging from 35.6% to 68.1% for user’s accuracy) and from 43.5% to 71% for producer’s accuracy). The producer’s accuracy of the Evergreen Broadleaf Forests, Deciduous Broadleaf Forests, and Mixed Forests was only 23.8%, 17.2%, and 10%, respectively. The user’s and producer’s accuracies of most land cover types under the CART classifier were visibly greater than the RF and SVM classifiers. Except for the producer’s accuracies of the Deciduous Broadleaf Forests, Mixed Forests, Barren, and Savannas, and the user’s accuracy of the Permanent Snow and Ice, the user’s and producer’s accuracies of other land cover types were the same and nearly 100%. In short, the user’s and producer’s accuracies of the CART in the training and validation samples were significantly better than the RF and SVM classifiers.

The overall accuracy and kappa coefficient are closely related to the overall classification performance. Table 3 is the accuracy assessment of training and validation samples. In the training samples, the overall accuracies of the RF and SVM classifiers were basically similar, and were 93.65% and 93.14%, respectively. The overall accuracy of the CART classifier was the greatest and reached 99.15%. Similarly, the kappa coefficient of the CART classifier was still greater than the RF and SVM classifiers. In the validation samples, the overall accuracy and kappa coefficient of the SVM classifier was lowest among the three kinds of classifiers. However, the overall accuracy and kappa coefficient of the CART classifier were visibly greater than the RF and SVM, indicating the CART classifier was more suitable to this automatic workflow for land cover classification and mapping.

**Table 3:** Accuracy assessment of training and validation samples.

Total Samples	Accuracy Assessment	RF	CART	SVM
**Training Samples**	Overall Accuracy	93.65%	99.15%	93.14%
	Kappa	0.93	0.99	0.93
**Validation Samples**	Overall Accuracy	74.36%	86.38%	66.87%
	Kappa	0.73	0.86	0.65

## 5. Discussion

Requirements for large volume data pre-processing and the collection of representative training samples are the most common difficulties encountered in supervised classification. Because the land cover types over a large study area are complex, mixed, and scattered (e.g., Evergreen Needleleaf Forests and Evergreen Broadleaf Forests, Deciduous Needleleaf Forests and Deciduous Broadleaf Forests, Closed Shrublands and Open Shrublands, etc.), the misclassification of land cover types results in generally low classification accuracy of remote sensing images. The present study documents a fast and reliable methodology to automatically classify large volume Sentinel-2 data using high-quality training samples derived from the MODIS land cover product. Here are the advantages (i) the land cover to be classified in an automated saving-manner without paying too much time and attention to collect and refine the accurate training data by visual interpretations; (ii) generation of a 20 m Sentinel land cover product with the same classification legend as 500 m MODIS land cover product; (iii) potential ability to provide global land cover mapping with reliable classification accuracy.

In this paper, the CART classifier had an overall accuracy and kappa coefficient of 86.38% and 0.86, respectively, visibly greater than values for RF and SVM classifiers. The user’s and producer’s accuracies of the Urban and Built-up Lands, Croplands, and Cropland/Natural Vegetation Mosaics in the training dataset were higher, probably because the spatial distribution of these land cover types in the study area was relatively concentrated. Further, the numbers of misclassified pixels under the RF classifier were obvious, for example, 15 pixels in the Permanent Wetlands were misclassified into the Permanent Snow and Ice, 17 pixels in the Grasslands were misclassified into the Cropland/Natural Vegetation Mosaics, and the Closed Shrublands had 20 and 22 pixels misclassified into the Croplands and Evergreen Broadleaf Forests, respectively (Fig 8A). There was a typical “salt-and-pepper” effect which was in similar spectral characteristics between Permanent Wetlands and Permanent Snow and Ice, Grasslands and Cropland/Natural Vegetation Mosaics, etc., because these land cover types were more refined [40,41]. Similarly, Hankui and David [28] assessed the level of agreement between the 30 m Landsat classifications and the MODIS land cover product-derived training data by bootstrapping the RF implementation. The overall accuracy and kappa coefficient of locally adaptive RF classification achieved 95.44% and 0.9443, respectively, which had a higher overall agreement than the single RF with 93.13% overall accuracy and 0.9195 kappa. The accuracy of the Sentinel-2 multispectral product used in this study is obviously different from the Landsat proposed by Hankui and David [28]. The overall accuracy and kappa of the RF classifier under the Sentinel-2 multispectral product were just 74.36% and 0.73, respectively, and lower than the Landsat, which showed the influence of remote sensing data on the classification accuracy.

The performance of classifiers is obviously different for land cover types [42]. In this study, the user’s and producer’s accuracies of the Cropland/Natural Vegetation Mosaics under the RF classifier were only 56.56% and 67.79%, respectively (Fig 8D), however, the user’s and producer’s accuracies under the CART classifier were 85.63% and 81.79%, respectively (Fig 8F). The user’s and producer’s accuracies of each land cover type under the RF, CART, and SVM classifiers were all different. Zhao et al. [43] compared the classification effects of different classifiers on land cover, and reported SVM classified rapidly but required detailed feature parameters; RF classifiers had fast speed, good stability, and highest accuracy but relatively poor stability. The overall accuracy and kappa coefficient of the SVM classifier were only 66.87% and 0.65, respectively, the lowest among the three kinds of classifiers. However, Fragou et al. [44] exploited the SVM classifier to classify the natural landscape in a Mediterranean environment using the Landsat Thematic Mapper images and found that overall accuracy was all-around 90%. Apparently, the performance of a certain classifier is influenced by the specific study area, the type of remote sensing images, the classification algorithms, the quantity and quality of training samples, and the dataset selected for accuracy assessment [45,46].

Further, ensemble methods can provide useful information like the Gini index to the end-user. The Gini index is a measure of statistical distribution intended to represent different attribute variables influencing the overall accuracy [47]. Using the Gini index, we were able to identify that B11 and B12 have a substantial effect on the overall model accuracy. Thus, using our proposed method, the classification performance can be increased by including additional indexes [48]. For example, including Normalized Difference Vegetation Index (NDVI) [49], Normalized Difference Salinity Index (NDSI) [50], and Normalized Difference Water Index (NDWI) [51] would help in getting a better performance.

## 6. Conclusions

Based on the self-programming in GEE cloud-based platform, a large and reliable training dataset of multispectral Sentinel-2 image was extracted automatically across the study area from the existing MODIS land cover product. To enhance confidence in high-quality training class labels, homogeneous 20 m Sentinel-2 pixels within each 500 m MODIS pixel were selected and a minority of heterogeneous 20 m pixels were removed based on calculations of spectral centroid and Euclidean distance. Further, the quality control and spatial filter were applied for all land cover classes to generate a reliable and representative training dataset that was subsequently applied to train the Classification and Regression Tree (CART), Random Forest (RF), and Support Vector Machine (SVM) classifiers. This approach generated a new Sentinel-2 land cover map for each classifier with the same legend as the MODIS product; The CART classifier appeared to be most suitable for this automatic workflow scheme, as its overall accuracy of 86.38% and kappa coefficient of 0.86 were greater than corresponding values for RF or SVM classifiers. The proposed method can automatically generate a large number of reliable and accurate training samples in a timely manner, which is promising for future land cover mapping in a large-scale region.

Moreover, the main land cover types in the study area as distinguished by three different classifiers were Evergreen Broadleaf Forests, Mixed Forests, Woody Savannas, and Croplands. The main land cover types in the northern part were Mixed Forests, Woody Savannas, with lesser areas classified as Croplands and Cropland/Natural Vegetation Mosaics. Areas classified as Evergreen Broadleaf Forests were mainly distributed in the eastern, western, and central parts of the study area. The southern part near the sea was dominated by the Urban and Built-up Lands, surrounded by a large area of Croplands and Cropland/Natural Vegetation Mosaics. Furthermore, there were scattered Permanent Wetlands in the southwest.

Finally, in the training and validation samples, the total pixel numbers (pixel size =  20 x 20 m) of the Evergreen Broadleaf Forests, Deciduous Broadleaf Forests, Mixed Forests, and Barren were the least (less than 17 pixels). Next, the total pixel numbers of the Woody Savannas and Water Bodies were nearly half the set value equal to 500 and 320 pixels. However, the total pixel numbers of the Open Shrublands and Grasslands were greater than 100 pixels but less than 150 pixels. The numbers of correctly classified pixels under the CART without computationally intensive were more than those for the RF and SVM classifiers. Moreover, the user’s and producer’s accuracies of the CART were significantly better than the RF and SVM classifiers. The overall accuracy and kappa coefficient of the CART classifier were the best. Furthermore, compared with the RF and SVM classifiers, the pixel percentages and overall spatial distribution of the land cover types were comparable under the CART classifier and MCD12Q1, indicating the CART classifier was more suitable to this automatic workflow for land cover mapping.
